# The Reviewer of Professor Dudley’s Surgical Essays

**Published:** 1850-01

**Authors:** 


THE REVIEWER OF PROFESSOR DUDLEY'S SURGICAL ESSAYS.

We are compelled to defer the response to the editor of the Transylvania Journal of Medicine, by the reviewer of Professor Dudley's essay on aneurism, whose review appeared in the November number of this Journal. This response shall appear in our next number.

